# Coupled hydromechanical and electromagnetic disturbances in unsaturated porous materials

**DOI:** 10.1002/wrcr.20092

**Published:** 2013-02-04

**Authors:** A Revil, H Mahardika

**Affiliations:** 1 Colorado School of Mines, Department of Geophysics, Green CenterGolden, Colorado, USA; 2 ISTerre, CNRS, UMR CNRS 5275, Université de SavoieLe Bourget du Lac, France

## Abstract

A theory of cross-coupled flow equations in unsaturated soils is necessary to predict (1) electroosmotic flow with application to electroremediation and agriculture, (2) the electroseismic and the seismoelectric effects to develop new geophysical methods to characterize the vadose zone, and (3) the streaming current, which can be used to investigate remotely ground water flow in unsaturated conditions in the capillary water regime. To develop such a theory, the cross-coupled generalized Darcy and Ohm constitutive equations of transport are extended to unsaturated conditions. This model accounts for inertial effects and for the polarization of porous materials. Rather than using the zeta potential, like in conventional theories for the saturated case, the key parameter used here is the quasi-static volumetric charge density of the pore space, which can be directly computed from the quasi-static permeability. The apparent permeability entering Darcy's law is also frequency dependent with a critical relaxation time that is, in turn, dependent on saturation. A decrease of saturation increases the associated relaxation frequency. The final form of the equations couples the Maxwell equations and a simplified form of two-fluid phases Biot theory accounting for water saturation. A generalized expression of the Richard equation is derived, accounting for the effect of the vibration of the skeleton during the passage of seismic waves and the electrical field. A new expression is obtained for the effective stress tensor. The model is tested against experimental data regarding the saturation and frequency dependence of the streaming potential coupling coefficient. The model is also adapted for two-phase flow conditions and a numerical application is shown for water flooding of a nonaqueous phase liquid (NAPL, oil) contaminated aquifer. Seismoelectric conversions are mostly taking place at the NAPL (oil)/water encroachment front and can be therefore used to remotely track the position of this front. This is not the case for other geophysical methods.

## 1. Introduction

*Revil et al*. [2011] recently developed a new set of constitutive equations to model cross-coupled transport phenomena in porous media. This model was however restricted to fully water saturated materials and for quasi-static flow problems (inertial effects were neglected). The motivation of the present paper is to extend the macroscopic generalized (cross-coupled) Darcy and Ohm laws to unsaturated porous materials and to account for dynamic effects (harmonic pressure or harmonic electrical fields and inertial terms). The final equations couple Biot's theory for unsaturated porous media to the Maxwell equations. The coupling is electrokinetic in nature.

There are broad applications of such a model for the assessment of water resources and remediation. For instance, the record of the electrical field of electrokinetic nature can be used to image nonintrusively ground water flow and to get access easily to the parameters characterizing the capillary pressure curve and the relative permeability [*Jougnot et al*., 2012; *Mboh et al*., 2012]. With a cross-coupled flow theory, it is also possible to model electroosmosis, which can be used to move solutes and nonaqueous phase liquids (NAPLs)/dense NAPLs (DNAPLs) in the vadose zone and aquifers for remediation purposes [*Acar and Alshawabkeh,* 1993; *Bruell et al*., 1992; *Han et al*., 2004]. This method can also be used to dewater clayey soils in civil engineering [*Bjerrum*, 1967]. Finally, electroosmosis can be used also to move moisture up to the roots of plants [*Huweg et al*., 2010], the required electrical field can be produced through the use of photovoltaics [*Kamel*, 1994].

The theory developed herein can be used to predict the electroseismic (electric-to-seismic conversion) effect in a broad range of frequencies (from 0.1 Hz to 100 kHz). The electroseismic coupling is related to the generation of seismic waves (hydromechanical disturbances) in response to a nonstationary electrical field [*Reppert and Morgan*, 2002; *Thompson*, 2005; *Thompson et al*., 2005]. While this effect has been mainly used so far for oil-related problems, it may be used to map NAPLs and DNAPLs as well [*Hinz*, 2011]. Alternatively, the seismoelectric (seismic-to-electric, SE) effect corresponds to the generation of electromagnetic disturbances from seismic waves [e.g., *Ivanov*, 1939; *Frenkel*, 1944; *Dupuis and Butler*, 2006; *Dupuis et al*., 2009; *Jardani et al*., 2010]. Hydromechanical disturbances (e.g., Haines jumps in unsaturated flow conditions, see *Haas and Revil* [2009]) can also be responsible for the generation of electromagnetic disturbances. These disturbances can in turn be remotely monitored by electromagnetic methods. To our knowledge, this is the first theory able to predict the electroseismic and SE effects in unsaturated conditions with applications to vadose zone hydrogeology. *Dupuis et al*. [2007] has demonstrated the feasibility to measure SE conversions for vadose zone applications.

## 2. Generalized Constitutive Equations

### 2.1. Assumptions

**Figure 1:**
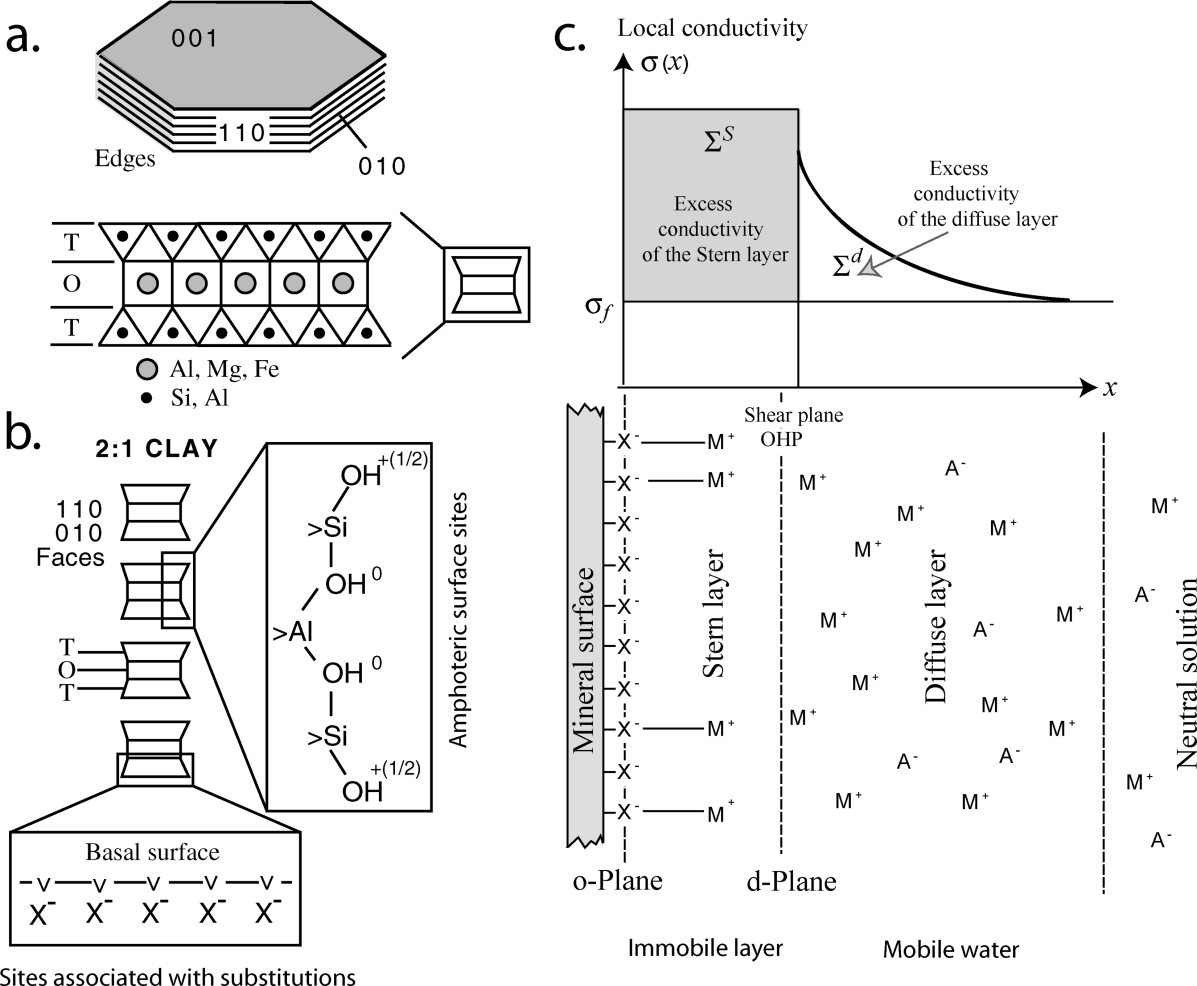
Double layer associated with clays. (a) TOT structure of clay minerals, where T stands for the tetrahedral layer (mainly Si) and O stands for the octohedral layer (mainly Al). (b) The surface of clay minerals in contact with water is charged because of both the existence of amphoteric sites on the edges of the clay crystal and basal negative sites associated with isomorphic substitutions in the crystalline framework of the clay minerals in the T layer and O layer. (c) The mineral charge is compensated by counterions (M^+^) and coions (A^−^) forming a double layer. This double layer comprises a layer of sorbed counterions (the Stern layer) and a diffuse layer where the Coulombic interactions between the charged mineral surface and the coions and counterions prevail. Note that a similar double layer exists at the surface of silica due to the reactivity of silanol sites with water. The parameter *f* denotes the fraction of the countercharge located in the Stern layer (partition coefficient).

We consider below an isotropic porous body with connected pores. The surface of the minerals in contact with water is negatively charged at near-neutral pH values. There is therefore an excess of electrical charges in the pore space. This excess of charges occurs in the electrical double layer (Figure 1). This electrical double layer is actually made up of two layers: (1) a layer of (counter) ions sorbed directly onto the mineral surface and (2) a diffuse layer. In the following, we will use the subscript *a* to describe the properties of air, and subscripts *w* and *s* for the water and solid phases, respectively. Water is assumed to be the wetting phase. The term skeleton (or frame) is used to describe the assemblage of grains alone without fluids in the pores.

Another set of assumptions used below pertain to the capillary pressure curve. Hysteretic behavior will be neglected, and therefore the porous material will thus be characterized by a unique set of hydraulic functions. In sections 1–5, we also work with the Richards model that assumes that the air pressure is constant and equal to the atmospheric pressure. This implies in turn that the air phase is infinitely mobile and connected to the atmosphere (in section 7, we will consider the case of two-phases flow using the approach of *Rubino and Holliger* [2012]). The capillary pressure 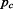 (in Pascal) is defined as the pressure of the nonwetting phase minus the pressure of the wetting phase [e.g., *Bear and Verruijt*, 1987],


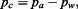
(1)

where 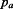 and 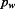 denote the average air and water pressures (in Pascal), respectively. The capillary head (in meter) is defined as


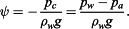
(2)

In unsaturated flow conditions, the gradient of the capillary head is given by


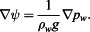
(3)

This assumption is used to avoid dealing with the flow of the air phase. In unsaturated conditions, the capillary pressure is positive, the capillary head (suction) is negative, and the pressure of the water phase is smaller than the atmospheric pressure. The total head includes the gravity force and is defined by 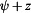, where *z* denotes the elevation. In the following, the model will be restricted to the capillary regime (saturation comprised between the irreducible water saturation and full saturation). Extending the present model below irreducible saturation would require a unified water capillary curve model (including a water sorption isotherm) and a description of film flow along the surface of the grains. This is outside the scope of the present paper.

Finally, attenuation of the seismic waves associated with squirt-flow dissipation mechanisms will be neglected despite the fact that this mechanism is known to control the attenuation of seismic waves in the frequency band usually used for seismic field investigations (see *Rubino and Holliger* [2012] for a recent pore-scale modeling of this effect and *Dvorkin et al*. [1994] and *Carcione* [2007] for a description of the relevant physics).

### 2.2. Generalized Darcy's Law

In saturated conditions, the (averaged) filtration displacement is defined as [e.g., *Morency and Tromp*, 2008],


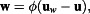
(4)

where **u** and **u***_w_* denote the averaged displacement of the solid and water phases, respectively, and *ϕ* denotes the connected porosity. A nomenclature of the material properties and mechanical properties used below is provided in Tables 1 and 2, respectively. The disturbances are considered to be harmonic of the form 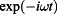, where 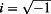 denotes the pure imaginary number, *ω* = 2*πf* denotes the angular frequency (in rad s^−1^), and *f* the frequency (in Hertz). In saturated conditions, the Darcy velocity is defined as the time derivative of the filtration displacement when the skeleton is moving. In water-saturated conditions, the generalized Darcy's law is given by [e.g., *Jardani et al*., 2010],


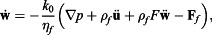
(5)

where 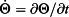 denotes the time derivative of the parameter 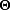 and *t* represents time, *k*_0_ denotes the low-frequency permeability of the porous material (in m^2^), 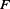 denotes the electrical formation factor, which is the ratio of the bulk tortuosity of the pore space to the connected porosity (see for instance, *Pride* [1994]), 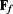 denotes the body force applied to the pore water phase (in N m^−3^, e.g., the gravitational body force or the electrical force acting on the excess of electrical charges of the pore water). The formation factor is related to the porosity by the first Archie's law 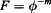, where *m* is called the cementation exponent [*Archie*, 1942]. To keep the notations as light as possible, we will not distinguish below if the variables are expressed in the time domain or in the frequency domain. It is easy to recognize if the equations are written in the frequency domain or in the time domain, the switch from one domain to the other being done by a Fourier transform or its associated inverse Fourier transform.

**Table 1:** Nomenclature of the Nonmechanical Material Properties

Symbol	Meaning	Unit
*F*	Formation factor	Dimensionless
*f*	Fraction of counterions in the Stern layer	Dimensionless
*m*	Cementation exponent	Dimensionless
*n*	Saturation exponent	Dimensionless
*λ*	Brooks and Corey exponent	Dimensionless
*r*	Coupling coefficient saturation exponent	Dimensionless
*q*	Characteristic time saturation exponent	Dimensionless
*k_S_*	Permeability at saturation	m^2^
*k*_0_	Low-frequency permeability	m^2^
*k_r_*	Relative permeability	Dimensionless
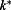	Complex permeability	m^2^
*K_h_*	Hydraulic conductivity	m s^−1^
*K_s_*	Hydraulic conductivity at saturation	m s^−1^
*C_S_*	Coupling coefficient at saturation	V m^−1^
*C*_0_	Low-frequency coupling coefficient	V m^−1^
*C_r_*	Relative coupling coefficient	Dimensionless
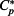	Complex streaming potential coupling coefficient	V m^−1^
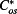	Electroosmotic coupling coefficient	C m^−3^
*ϕ*	Porosity	Dimensionless
*σ*	Electrical conductivity	S m^−1^
*σ′*	In-phase electrical conductivity	S m^−1^
*σ″*	Quadrature conductivity	S m^−1^
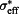	Complex effective electrical conductivity	S m^−1^
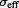	(Real) effective electrical conductivity	S m^−1^
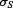	Surface conductivity of the solid phase	S m^−1^
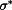	Complex electrical conductivity	S m^−1^
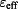	(Real) effective dielectric constant	F m^−1^
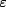	Dielectric constant	F m^−1^
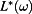	Streaming current coupling coefficient	A m^−2^
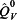	Moveable charge density at low frequency	C m^−3^
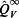	Moveable charge density at high frequency	C m^−3^
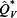	Complex moveable charge density	C m^−3^
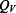	Total charge density from the CEC	C m^−3^
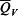	Charge density of the diffuse layer	C m^−3^
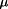	Magnetic permeability	H m^−1^

**Table 2:** Nomenclature of the Mechanical Material Properties

Symbol	Meaning	Unit
*C*	Biot modulus	Pa
*C_s_*	Biot modulus at saturation	Pa
*M*	Biot modulus	Pa
*M_s_*	Biot modulus at saturation	Pa
*α*	Biot coefficient at saturation	Dimensionless
*α_w_*	Biot coefficient at partial saturation	Dimensionless
*G=G_fr_*	Shear modulus of the solid frame	Pa
*K=K_fr_*	Bulk modulus of the solid frame	Pa
*λ*	Lamé coefficient	Pa
*λ_u_*	Undrained Lamé coefficient	Pa
*K_a_*	Bulk modulus of the air	Pa
*K_o_*	Bulk modulus of the oil	Pa
*K_w_*	Bulk modulus of water	Pa
*K_S_*	Bulk modulus of the solid phase	Pa
*K_f_*	Bulk modulus of the fluid phase	Pa
*K_u_*	Undrained bulk modulus	Pa

In unsaturated conditions, the filtration displacement and the mass density of the water phase are given by,


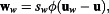
(6)


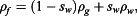
(7)

respectively, 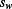 denotes the water saturation (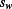 = 1 at saturation). The mass density of the gas phase can be neglected, and therefore 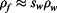. From equation (4), the porosity can be replaced by 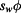 when dealing with unsaturated conditions (of course, terms in (1 − *ϕ*), dealing with the solid phase, remain unchanged). The Darcy velocity associated with the water phase is given by,


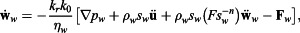
(8)

where 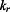 denotes the relative permeability (dimensionless), 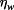 denotes the dynamic viscosity of the pore water (in Pa s), and *p_w_* is the pressure of the water phase (it will be replaced later by the suction head, see equation (3) above). The term 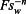 denotes the ratio of the bulk tortuosity of the water phase divided by the connected porosity, *n* denotes Archie's second or saturation Archie's exponent (*n* > 1, dimensionless, *Archie* [1942]).

From now, the constitutive equations are described in the frequency domain and 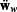 in the right-hand side of equation (8) can be replaced by 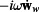 and combined with the 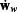 term found in the left-hand side of equation (8). Equation (8) can then be rewritten as,


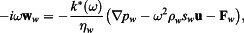
(9)

where 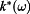 is a complex-valued apparent permeability given by


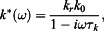
(10)

with the relaxation time 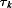 given by,


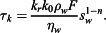
(11)

The complex permeability is used to describe the effect of the inertial force in Darcy's law [e.g., *Pride*, 1994]. In the time domain, a nonlinear Darcy's law can be similarly defined in which the apparent permeability depends on the Reynolds number (see *Bolève et al*. [2007] for the development of the equations and an experimental check). Alternatively, this equation could be written to be consistent with the Forchheimer equation. In the Forchheimer equation, the flux is decomposed in several terms while in the equations derived in *Bolève et al*. [2007], this is the right-hand side of the Darcy equation that is developed as a nonlinear function of the fluid pressure (nonlinear Darcy equation). *Aulisa et al*. [2009] derived a straightforward approach to go from one formulation to the other. The relaxation time 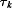 characterizes the transition between the viscous laminar flow regime and the inertial laminar flow regime in the Navier-Stokes equation formulated in the frequency domain. The critical frequency associated with this relaxation time is given by,


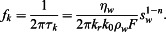
(12)

Note that because *n* ≥ 1 [*Archie*, 1942], 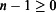. The measurement of this relaxation frequency can be used to estimate the (low-frequency) permeability of the material.

**Figure 2:**
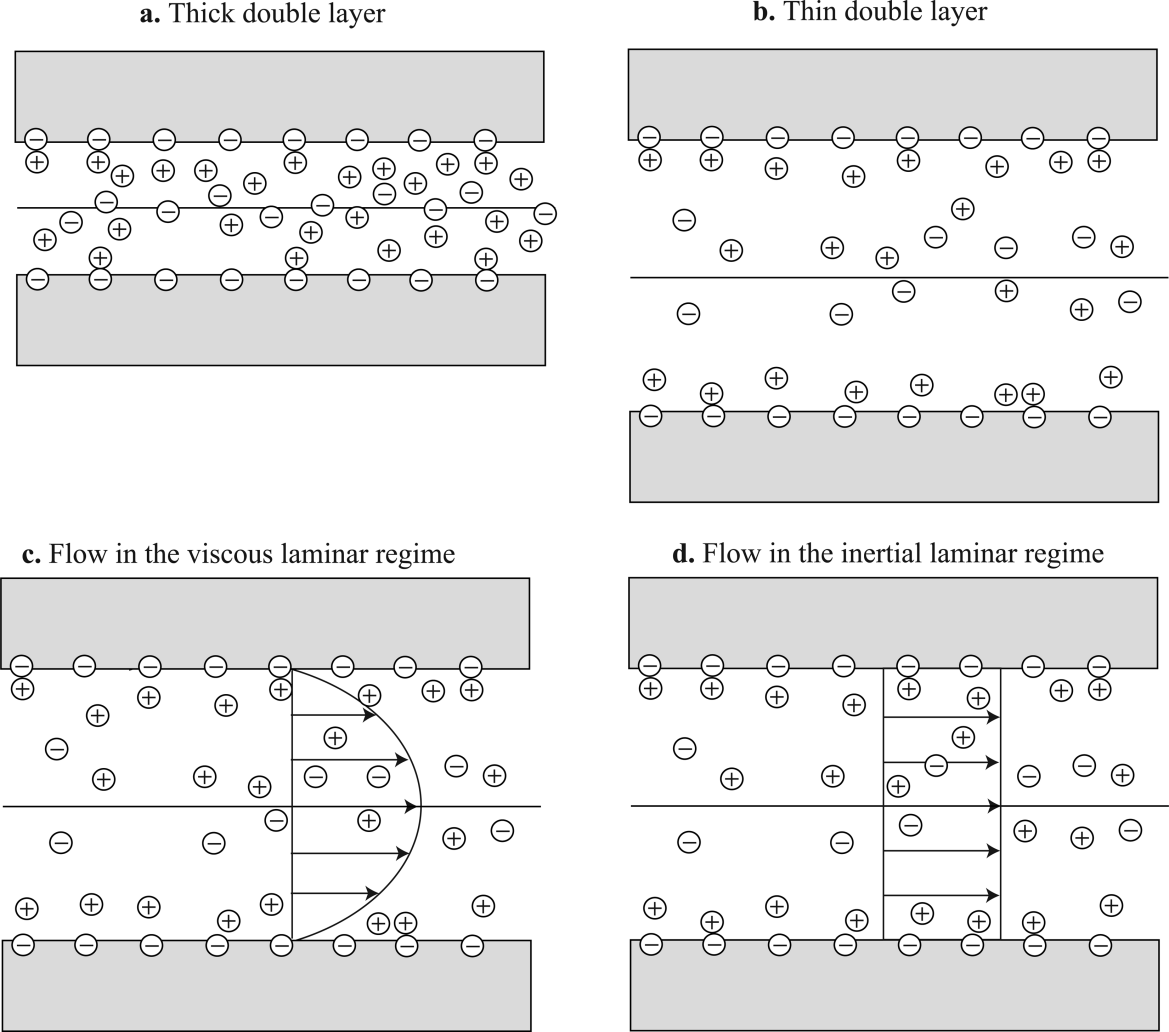
Sketch of the charge distribution and flow regime in a pore. There are four end members to consider, depending on the pore size with respect to the double-layer thickness and on the frequency. (a) Thick double layer. The counterions of the diffuse layer are uniformly distributed in the pore space). (b) Thin double layer (the thickness of the diffuse layer is much smaller than the size of the pores). (c) Viscous laminar flow regime occurring at low frequencies. (d) Inertial laminar flow regime occurring at high frequencies.

The two-flow regimes in porous media are sketched in Figure 2. The low-frequency flow regime (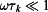, 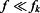) corresponds to the viscous laminar flow regime for which the flow in a cylindrical pore obeys Poiseuille's law (Figure 2c). High frequencies (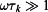, 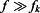) corresponds to the inertial laminar flow regime for which the pore water flow is described as being a potential-flow problem. We consider here Reynolds number that are much below the values corresponding to the turbulent regime.

In poroelasticity, it is customary to define the following two variables [e.g., *Morency and Tromp*, 2008; *Jardani et al*., 2010, and references therein],


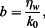
(13)


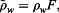
(14)

where 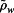 denotes an apparent pore water mass density. The relationship between the permeability and the water saturation can be expressed with the *Brooks and Corey* [1964] relationship,


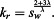
(15)

From equation (11) and equations (13)–(15), we obtain,


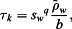
(16)

where 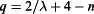. In *Revil* [2013, Appendix A], it is shown that 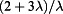 scales approximately as (*n*+2), so 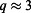.

In order to write a hydrodynamic equation coupled with the electrical field, the body force 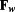 entering equation (9) should be expressed by Coulomb's law,


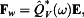
(17)

where 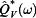 denotes the frequency-dependent excess of charge that can be dragged by the flow of the pore water through the pore space of the material (dynamic excess charge density of the pore space, see Appendix A) and **E** denotes the electrical field (in V m^−1^). The charge density 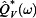 is frequency dependent because there is more charges dragged in the inertial laminar flow regime than in the viscous laminar flow regime in agreement with the model of *Pride* [1994]. In the following, the parameters 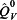 and 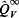 are the (dynamic or effective) volumetric charge density dragged in the low- frequency (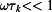) and high-frequency (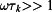) regimes, respectively. Because the transition between low-frequency and high-frequency regimes is governed by the relaxation time 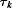, the following functional can be used to compute the effective charge density as a function of the frequency (Appendix A):


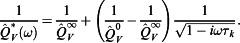
(18)

The low-frequency and high-frequency charge densities 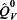 and 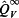 can be found as follows.

(1) At low frequencies only a small fraction of the counterions of the diffuse layer are dragged by the flow of the pore water and therefore 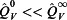. An expression to compute 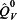 directly from the low-frequency permeability 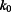 is discussed further in section 5a below.

(2) At high frequencies, all the charge density existing in the pores is uniformly dragged along the pore water flow, and therefore the charge density 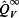 is also equal to the volumetric charge density of the diffuse layer. An expression to compute 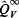 from the cation exchange capacity (CEC) is discussed further in section 5b below.

Depending on the size of the electrical double layer with respect to the size of the pores, two cases need to be considered:

(1) In the thick double-layer approximation (see Figure 2a), 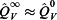 (all the counterions of the diffuse layer are dragged by the flow whatever the frequency), and therefore


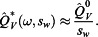
(19)

(2) In the thin double layer approximation (see Figure 2b), one can expect 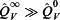 (see examples in *Jougnot et al*. [2012] and discussion in section 5 below) and therefore (see Appendix A)


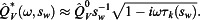
(20)

Introducing Coulomb's law, equation (17), into the Darcy equation, equation (9), yields,


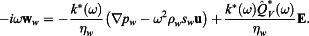
(21)

Equation (21) shows the influence of three forcing terms on the Darcy velocity: (i) the displacement of the solid framework (through viscous drag at the pore water/solid interface), (ii) the pore fluid pressure gradient (in which a gravitational component can be added if needed), and (iii) the electrical field (which corresponds to a body force per unit charge density) through electroosmosis. Electroosmosis refers to the flow of the pore water in response to an electrical field. Physically, the positive excess of charge is responsible for a net flow in the direction of the electrical field through viscous drag of the pore water by the excess of electrical charge in the pore water.

### 2.3. Generalized Ohm's Law

We investigate now the macroscopic electrical current density **J**. The first contribution is the conduction current density 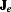 given by Ohm's law,


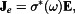
(22)

where the conductivity 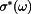 denotes the complex conductivity. For clayey materials, this conductivity has been modeled recently by *Revil* [2012, 2013]. The frequency dependence of the apparent conductivity is a direct consequence of the fact that the force applied to the charge carriers is controlled by an electrochemical potential, not just by the electrical field. Therefore, electromigration and diffusion are always coupled phenomena in porous media. The second contribution to the total current density corresponds to the advective drag of the excess of charge of the pore space by the flow of the pore water (contribution of advective nature). If the Darcy velocity associated with the poromechanical contribution is written as 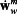, the second contribution to the current density (source current density) is given by (see Appendix A),


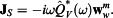
(23)

The mechanical contribution to the filtration displacement is given by the generalized Darcy's law derived in section 2.2 above,


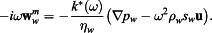
(24)

Equations (22)–(24) yield the following generalized Ohm's law,


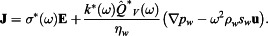
(25)

A model for the complex conductivity is now discussed. The complex conductivity is written as,


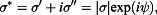
(26)

where 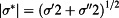 denotes the magnitude of the conductivity and 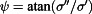 denotes the phase lag between the electrical current and the resulting electrical field. For clayey materials, the complex conductivity is observed to be relatively independent on the frequency in the range 0.1 Hz–10 kHz [see *Vinegar and Waxman*, 1984; *Revil*, 2012, 2013]. The in-phase electrical conductivity is given as a function of the pore water conductivity 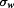 by,


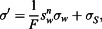
(27)

where *F* denotes the formation factor introduced above and 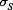 denotes the surface conductivity. The surface conductivity is given by the model developed by *Revil* [2012, 2013]:


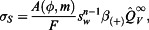
(28)


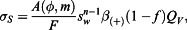
(29)

where *A*(*ϕ*, *m*) (∼1) is defined by,


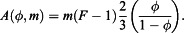
(30)

An alternative expression is given by *Revil* [2013].

Equation (29) implies that the surface conductivity is controlled by the diffuse layer containing the fraction of counterions (1 − *f*) (Figure 1) characterized by an ionic mobility 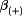 equal to the mobility of the cations in the bulk pore water [*Revil*, 2012, 2013].

Following *Revil* [2012, 2013], the quadrature conductivity can be expressed as,


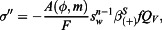
(31)


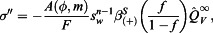
(32)

where 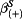 denotes the mobility of the counterions in the Stern layer (see value in *Revil* [2012, 2013]). These equations provide a simple and accurate model to describe the complex conductivity of shaly sands and soils and generalize the *Vinegar and Waxman* [1984] model. As noted by *Vinegar and Waxman* [1984] and *Revil* [2012], the frequency dependence of the quadrature conductivity is not explicit in equation (32). That said, for quasi-static conditions, the quadrature conductivity should go to zero under DC conditions,


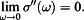
(33)

The typical frequency below which the quadrature conductivity becomes frequency dependent is typically smaller than 0.1 Hz [see *Revil*, 2012] and is therefore not relevant to the SE and SE problems. Note thus that the present approach includes a complex conductivity that agree with experimental data, while the frequency dependence of the complex conductivity in *Pride* [1994] is due to an electroosmotic contribution that is expected to be negligible (see discussion in *Marshall and Madden* [1959]) and therefore cannot explain the observed quadrature conductivity of porous clayey materials (note that *Pride* [1994] stated very clearly in his paper that induced polarization was neglected in his approach).

### 2.4. Coupled Constitutive Transport Equations

The two constitutive equations for the generalized Ohm and Darcy laws are written into the following matrix form:


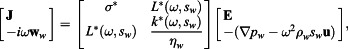
(34)

where the cross-coupling term 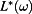 is defined as,


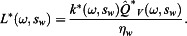
(35)

The generalized streaming potential coupling coefficient is defined by the following equations in the quasi-static limit of Maxwell equations:


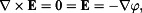
(36)


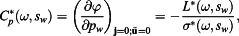
(37)


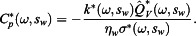
(38)

More explicitly, in the thin-double layer approximation, the dynamic streaming potential coupling coefficient is given by,


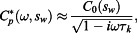
(39)


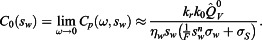
(40)

Equation (40) (developed by Revil and coworkers, see *Revil et al*. [2007] and *Linde et al*. [2007] for quasi-static streaming potentials) has been successfully tested by *Mboh et al*. [2012] through laboratory measurements. A check of this model will be provided below in section 6.

Similarly, the generalized electroosmotic coupling coefficient is defined in the quasi-static limit of the Maxwell equations and when the skeleton is at rest and in absence of pore fluid flow by the ratio between the gradient of pore fluid pressure divided by the gradient in electrical potential. For the thin-double layer case, this yields,


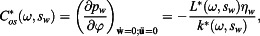
(41)


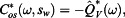
(42)


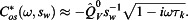
(43)

For the case corresponding to the thick double layer, the electroosmotic coupling coefficient is given by 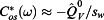. Therefore, the electroosmotic coupling coefficient is simply a measure of the excess of charges that can be moved by the flow of the pore water and that this excess of charge increases with the frequency between the viscous laminar flow and the inertial laminar flow regime. It also increases with the decrease of the water saturation. Note however that in equation (34), this is 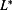 (not 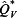) that controls the magnitude of the Darcy velocity, and therefore the intensity of the electroosmotic contribution to the Darcy velocity decreases with the saturation, which is qualitatively in agreement with the observations made by *Aggour et al*. [1994].

In unsaturated flow conditions, it is customary to use the capillary head gradient 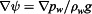 instead of the pore water pressure gradient (see section 2.1 above). Below the relaxation frequency separating the low-frequency viscous laminar flow regime from the high-frequency inertial laminar flow regime (true in the frequency range used for field applications), equation (34) can be rewritten in the time domain using the pressure head and including the gravitational field in the hydraulic driving force. This yields,


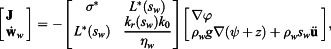
(44)

where the quasi-static coupling coefficient 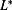 is given by,


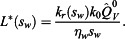
(45)

Equations (44) and (45) therefore provide a simple model for modeling the occurrence of streaming potential and electroosmosis in porous media.

## 3. Description of the Hydromechanical Model

### 3.1. Generalized Diffusion Equation for the Pore Water

The starting point is the following Biot constitutive equation in saturated conditions:


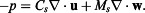
(46)

Equation (46) is also often written as


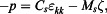
(47)

where 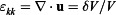 (where *V* denotes the volume of the representative elementary volume) denotes the volumetric strain of the porous body and 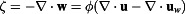 denotes the linearized increment of fluid content [e.g., *Lo et al*., [2002]. The parameter 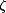 represents the fractional volume of water flowing in or out of the representative volume of skeleton in response to an applied stress. The bulk moduli 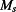 and 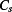, in saturated conditions, are defined as [e.g., *Pride*, 1994],


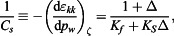
(48)


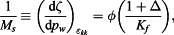
(49)

where 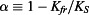 denotes the Biot coefficient in the saturated state and 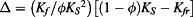. The Biot modulus *M* corresponds to the inverse of the poroelastic component of the specific storage and is defined as the increase of fluid (per unit volume of rock) as a result of an increase of pore pressure under constant volumetric strain. The following relationship applies 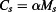.

To extend these equations to the unsaturated case, we apply the classical change of variables used in section 2 above (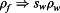, 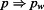, 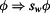, and 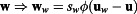). This yields


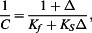
(50)


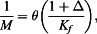
(51)

where 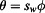 denotes the water content (dimensionless) and where, in unsaturated conditions, the fluid increment is defined by [*Berryman et al*., 1988],


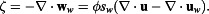
(52)

The term 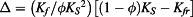 depends on the saturation because the compressibility of the fluid is given for instance by the Wood formula 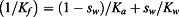 [*Wood*, 1955; *Teja and Rice*, 1981], where 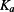 and 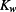 represent the bulk moduli of air and water, respectively (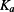 = 0.145 MPa and 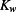 = 2.25 GPa, see *Lo et al*. [2005])).

In unsaturated conditions, from equations (50) and (51), 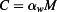 and the Biot coefficient 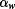 is given by 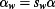, where *α* denotes the Biot coefficient in saturated conditions. That said, there should be no exchange of water below the irreducible water saturation and therefore the correct scaling should be 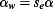 rather than 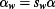, where 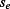 denotes the reduced water saturation 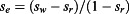 where *s_r_* denotes the irreducible water saturation. In other words 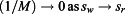.

In addition to the poroelastic change corresponding to equation (51), there is also a change related to the moisture capacity. Generalizing equation (46) to unsaturated conditions yields therefore,


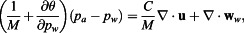
(53)

where 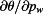 denotes the specific moisture capacity which is determined from the derivative of the capillary pressure with respect to the water content (in unsaturated flow, the air pressure is kept constant and close to saturation 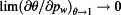). From section 2a, the filtration displacement of the water phase is given by


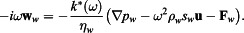
(54)

Therefore, the filtration displacement is given by,


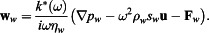
(55)

Equations (53) and (55) yield,


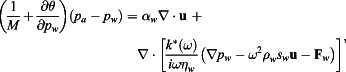
(56)

where the relationship 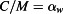 has been used. Equation (56) is a nonlinear diffusion equation for the fluid pressure. For this to be obvious, the terms of this equation need to be reworked. Multiplying all the terms by 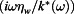, separating the pressure terms in the left-hand side from the source term in the right-hand side and taking into consideration that the air pressure is constant (unsaturated flow assumption), the following nonlinear hydraulic diffusion equation is obtained,


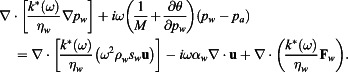
(57)

Equation (57) can be written in the time domain using an inverse Fourier transform. Assuming that the permeability is given by its low-frequency asymptotic limit (which is correct below 10 kHz) and using Coulomb law plus the gravity force as body force (the frequency-dependent volumetric charge density is also taken in its low frequency limit too) yield,


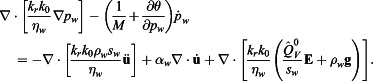
(58)

The origin of the three forcing terms on the right-hand side of this equation is now clearly established: the first term is related to the acceleration of the seismic wave acting on the skeleton of the material, the second term is due to the velocity of the seismic wave, and the third term (at constant gravity acceleration) corresponds to the pore water flow associated with the electroosmotic forcing associated with the drag of the pore water by the electromigration of the excess of charge contained in the pore space of the material.

Another possibility is to write a generalized Richards equation [*Richards*, 1931] showing the influence of the forcing terms in this equation (we assume again that the air pressure is constant). Starting with equation (58) and replacing the water pressure by the capillary head defined by 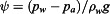 (in meter), the following Richards equation is obtained,


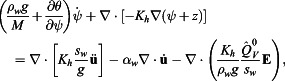
(59)


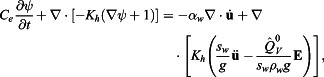
(60)


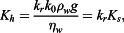
(61)

where 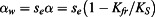 and where 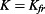 denotes the bulk modulus of the skeleton (drained bulk modulus), *K_h_* denotes the hydraulic conductivity (in m s^−1^), 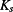 denotes the hydraulic conductivity at saturation, and *C_e_* denotes the specific storage term. This storage term is the sum of the specific moisture capacity (in m^−1^) (also called the water capacity function) and the specific storage corresponding to the poroelastic deformation of the material. This yields,


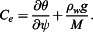
(62)

Usually in unsaturated conditions, the poroelastic term is much smaller than the specific moisture capacity, but the poroelastic term should be kept to have a formulation that remains consistent with the saturated state. The hydraulic conductivity is related to the relative permeability 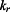 and 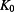, the hydraulic conductivity at saturation. With the *Brooks and Corey* [1964] model, the porous material is saturated when the fluid pressure reaches the atmospheric pressure (*ψ* = 0 at the water table). The effective saturation, the specific moisture capacity, the relative permeability, and the water content are defined by


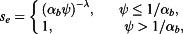
(63)


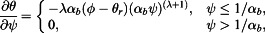
(64)


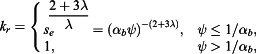
(65)


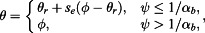
(66)

respectively, where 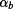 denotes the inverse of the capillary entry pressure related to the matric suction at which pore fluid begins to leave a drying porous material, *λ* is called the pore size distribution index (a textural parameter), 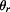 represents the residual water content (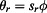). Sometimes the residual water saturation is not accounted for and the capillary pressure curve and the relative permeability are written as,


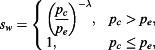
(67)


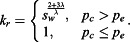
(68)

Because 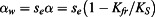, when the water saturation reaches the irreducible water saturation, the two source terms on the right-hand side of equation (60) are null. Therefore, there is no possible excitation below the irreducible water saturation. In reality, this is not necessarily true, and the model could be completed by including film flow below the irreducible water saturation.

### 3.2. Newton's Law and the Definition of the Effective Stress

The hydromechanical equations are defined in terms of an effective stress tensor. As explained in detail by *Jardani et al*. [2010] and *Revil and Jardani* [2010], there is a computational advantage in expressing the coupled hydromechanical problem in terms of the fluid pressure and displacement of the solid phase (four unknowns in total) rather than using the displacement of the solid and filtration displacement (six unknowns in total).

In saturated conditions, Newton's law (which is a momentum conservation equation for the skeleton partially filled with its pore water) is written as,


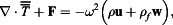
(69)

where 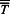 is the total stress tensor (positive normal stress for tension, see *Detournay and Cheng* [1993]) and **F** denotes the total body force applied to the porous material. In unsaturated conditions, Newton's law is written as,


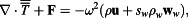
(70)

where the filtration displacement of the pore water phase is given by,


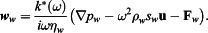
(71)

Combining equations (70) and (71) yields,


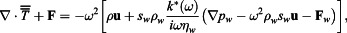
(72)


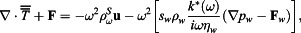
(73)

where,


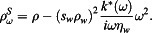
(74)

The effective stress in unsaturated conditions is taken as (this choice is justified below),


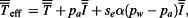
(75)

which is both consistent with Bishop effective stress principle in unsaturated conditions and the Biot stress principle in saturated conditions. The confining pressure and the effective confining pressure are defined as,


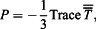
(76)


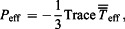
(77)

respectively. This yields 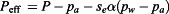 in agreement with the recent results by *Lu et al*. [2010]. Equations (73) and (75) yield,


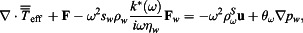
(78)

where the hydromechanical coupling term 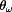 is defined by


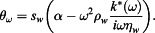
(79)

The last fundamental constitutive equation needed to complete the hydromechanical model in unsaturated conditions is a relationship between the total stress tensor (or the effective stress tensor) and the displacement of the solid phase and filtration displacement of the pore water phase. This equation is Hooke's law, which, in linear poroelasticity and for saturated conditions, is given by


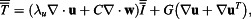
(80)

where the infinitesimal deformation tensor is related to the displacement of the solid phase by 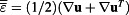, 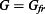 denotes the shear modulus that is equal to the shear modulus of the skeleton (frame), and 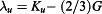 denotes the Lamé modulus in undrained conditions (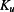 denotes the undrained bulk modulus). In unsaturated conditions and accounting for the air pressure, equation (78) can be written as,


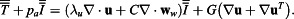
(81)

From equation (53) of section 3.1 above, the linearized increment of fluid content is given by,


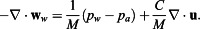
(82)

Combining equations (81) and (82) yields,


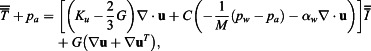
(83)


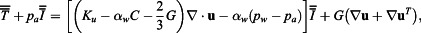
(84)

where the following expression derived in section 3.1 has been used for the Biot coefficient in unsaturated conditions,


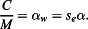
(85)

In addition, the bulk modulus is given by 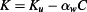 and the Lamé Modulus is given by 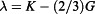. Equation (84) yields the following Hooke's law for the effective stress


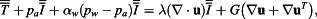
(86)


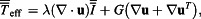
(87)

where the effective stress is also given by equation (75). Note that the effective stress is only related to the deformation of the skeleton of the porous material (by definition). This model generalizes therefore the effective stress concept developed recently by *Lu et al*. [2010].

## 4. Description of Maxwell Equations

*Pride* [1994] volume averaged the local Maxwell equations to obtain a set of macroscopic Maxwell equations in the thin-double layer limit. The same equations were obtained by *Revil and Linde* [2006] for the thick double-layer case. The form of the four macroscopic Maxwell equations (Faraday's law of induction, Ampère's law, Gauss's law for magnetism, Gauss's law) is,


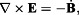
(88)


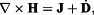
(89)


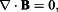
(90)


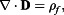
(91)

where 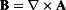 denotes the magnetic field (in T), **H** is the auxiliary magnetic field (in A m^−1^), and **D** is the current displacement vector (in C m^−2^), **A** is the magnetic (vector) potential, 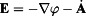, where *φ* denotes the electric (scalar) potential (in V), and 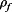 denotes the density of free charges These equations are completed by two electromagnetic constitutive equations: 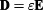 and 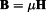, where *ε* is the permittivity of the porous body and *µ* denotes its magnetic permeability. In the absence of magnetized grains, 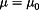 where 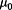 denotes the magnetic permeability of free space.

When the harmonic electrical field is written as 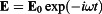, and *ω* is the angular frequency with **E**_0_ a constant electrical field magnitude and direction, the displacement current density vector is given by 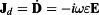. The total current density 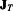 entering Ampere's law,


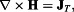
(92)

is given by,


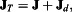
(93)


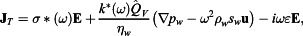
(94)


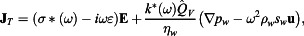
(95)

and an effective conductivity can be introduced such as 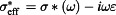, where 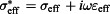 is the effective or apparent complex conductivity and 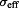 and 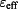 are real scalars dependent upon frequency. These effective parameters are the parameters that are measured during an experiment in the laboratory or in the field and contain both electromigration and true dielectric polarization effects. They are given by 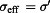 and 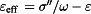. Another recently published paper is dedicated to the description of these parameters in the frequency range 1 mHz–1 GHz [*Revil*, 2013].

## 5. Determination of Charge Densities

The goal of this section is to provide a way to estimate the two charge densities 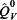 and 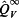 used in the previous sections. We first start by the low-frequency charge density and then we provide a model to estimate the high-frequency charge density.

### 5.1. Determination of the Quasi-Static Excess Charge Density

For a fully water saturated material, the streaming potential coupling coefficient is defined as,


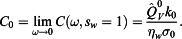
(96)

Equation (96) provides a way to estimate the charge density 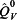 from the measurements of the low-frequency streaming potential coupling coefficient, the low-frequency electrical conductivity, and permeability using,


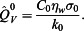
(97)

**Figure 3:**
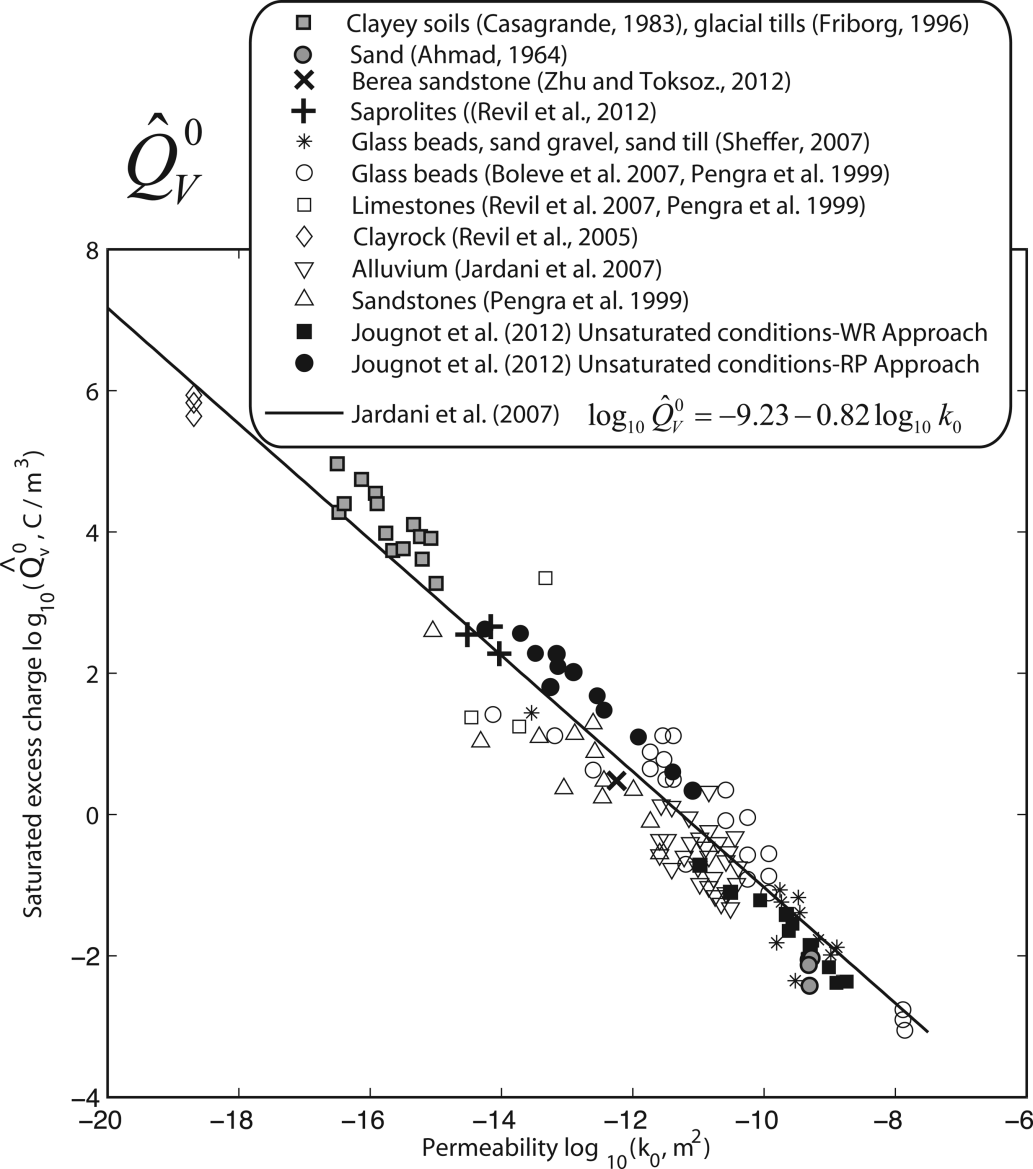
Quasi-static charge density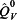 (excess pore charge moveable by the quasi-static pore water flow) versus the quasi-static permeability for a broad collection of core samples and porous materials. This charge density is directly derived from the streaming potential coupling coefficient using equation (97). Data from *Ahmad* [1969], *Bolève et al*. [2007], *Casagrande* [1983], *Friborg* [1996], *Jougnot et al*. [2012], *Jardani et al*. [2007], *Pengra et al*. [1999], *Revil et al*. [2012, 2007], *Sheffer* [2007]; *Revil et al*. [2013], and *Zhu and Toksöz* [2012].

The estimate of the low-frequency volumetric charge density 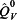 is reported as a function of the permeability in Figure 3 for experiments performed with a broad range of porous materials at near-neutral pH values (pH 5–8). According to *Jardani et al*. [2007], 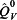 can be directly estimated from the quasi-static (saturated) permeability by (see Figure 3):


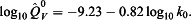
(98)

Equation (98) therefore provides a way to estimate directly the volumetric charge density from the low-frequency permeability, thereby reducing the number of material properties to consider in the simulations.

### 5.2. Determination of the High-Frequency Excess Charge Density

We now seek a way to estimate the high-frequency charge density 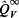. At full saturation, the surface conductivity is given by,


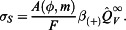
(99)

Therefore, the determination of the surface conductivity and the formation factor (from the measurements of the conductivity of the porous material at different pore water conductivities) can be used to assess the value of 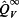. Therefore, the high-frequency excess charge density 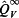 can be estimated to the surface conductivity and the formation factor by,


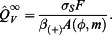
(100)

**Figure 4:**
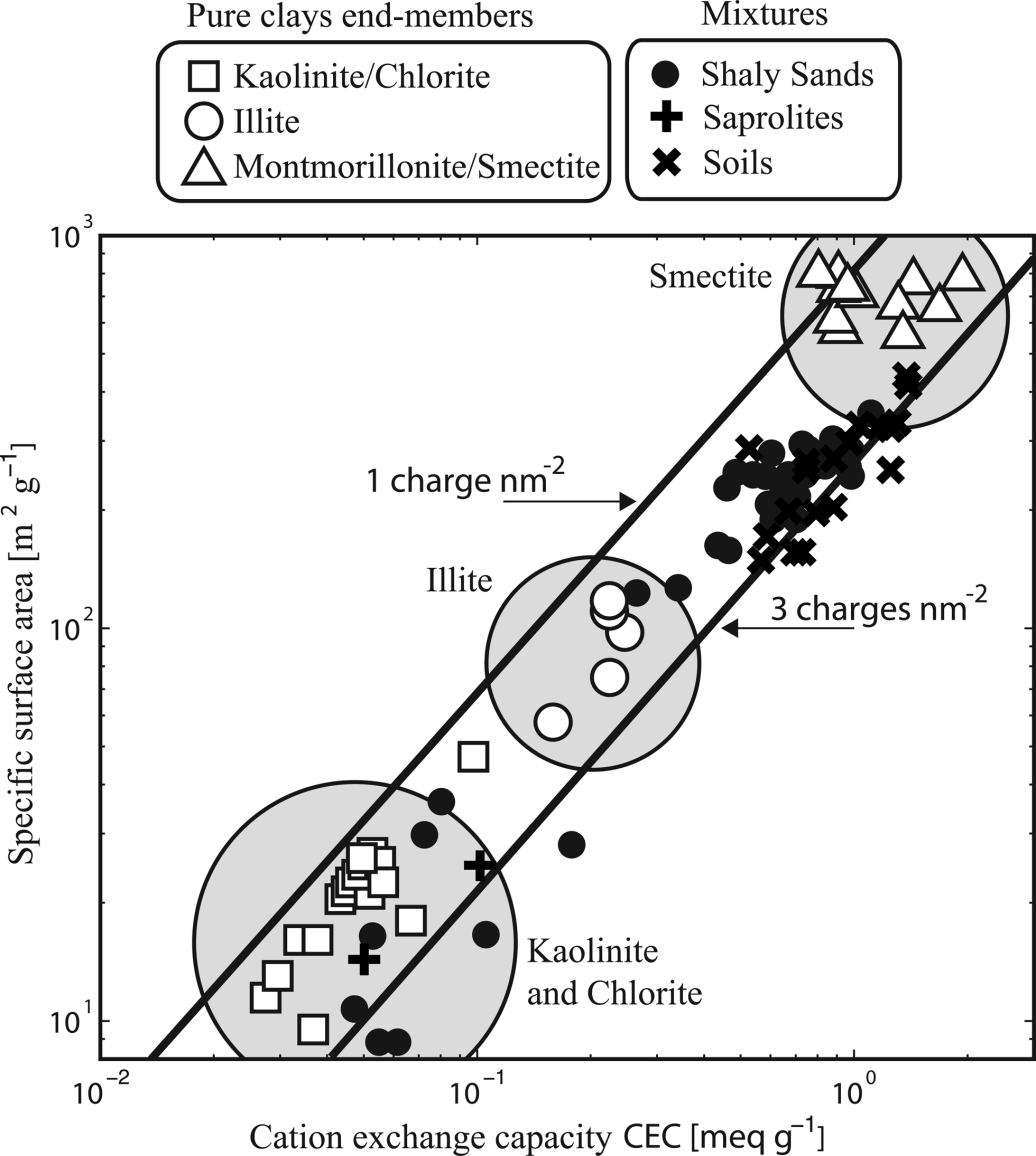
Specific surface area of clay minerals *S_s_* (in m^2^ g^−1^) as a function of the CEC (in meq g^−1^ with 1 meq g^−1^=96,320 C kg^−1^ in SI units) for various clay minerals. The ratio between the CEC and the specific surface area gives the equivalent total surface charge density of the mineral surface. The shaded circles correspond to generalized regions for kaolinite, illite, and smectite. Figure adapted from *Revil and Leroy* [2004]. The two lines corresponds to one to three elementary charges per unit surface area. Data for the clay end members are from: *Patchett* [1975], *Lipsicas* [1984], *Zundel and Siffert* [1985], *Lockhart* [1980], *Sinitsyn et al*. [2000], *Avena and De Pauli* [1998], *Shainberg et al*. [1988], *Su et al*. [2000], and *Ma and Eggleton* [1999]. Saprolite data: *Revil et al*. (2013). Soil data: *Chittoori and Puppala* [2011].

Indeed, at high frequencies, all the charge density existing in the pores is uniformly dragged along the pore water flow, and therefore the charge density 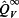 is also equal to the volumetric charge density of the diffuse layer [*Revil and Florsch*, 2010],


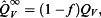
(101)

where *f* is the fraction of counterions in the Stern layer and 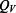 denotes the total charge density in the pore space including the contribution of the Stern layer. This total charge density (Stern plus diffuse layers) is related to the CEC (in mol kg^−1^) by [*Waxman and Smits*, 1968],


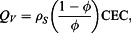
(102)

where 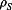 denotes the mass density of the grains. The CEC is another fundamental parameter describing the electrochemical properties of the porous material, more precisely the amount of active surface sites on the mineral surface at a given pH value. In SI units, the CEC is expressed in C kg^−1^, but is classically expressed in meq g^−1^ (with 1 meq = 1 mmol equivalent charge, e.g., 1 × 10^−3^ *e N*, where *e* = 1.6×10^−19^ C and *N* is the Avogadro number, 6.022 × 10^23^ mol^−1^, 1 meq g^−1^ = 96,320 C kg^−1^). The fraction of counterions *f* can be determined from electrical double-layer theory [see *Revil and Florsch*, 2010]. For materials with different types of clay minerals, the average CEC is determined from the respective exchange capacities of the constituent clay types using [*Rabaute et al*., 2003; *Woodruff and Revil*, 2011],


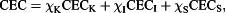
(103)

where 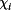 represents the mass fraction of mineral *i* in the porous material and K, I, and S stand for kaolinite, illite, and smectite, respectively, in the clayey material. The CEC of the clay members are well tabulated (see Figure 4). If there is only one type of clay mineral, the CEC is given by 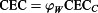, where 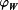 denotes the clay fraction (in weight) of the porous material and 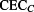 denotes the CEC of the clay minerals present in the material.

**Figure 5:**
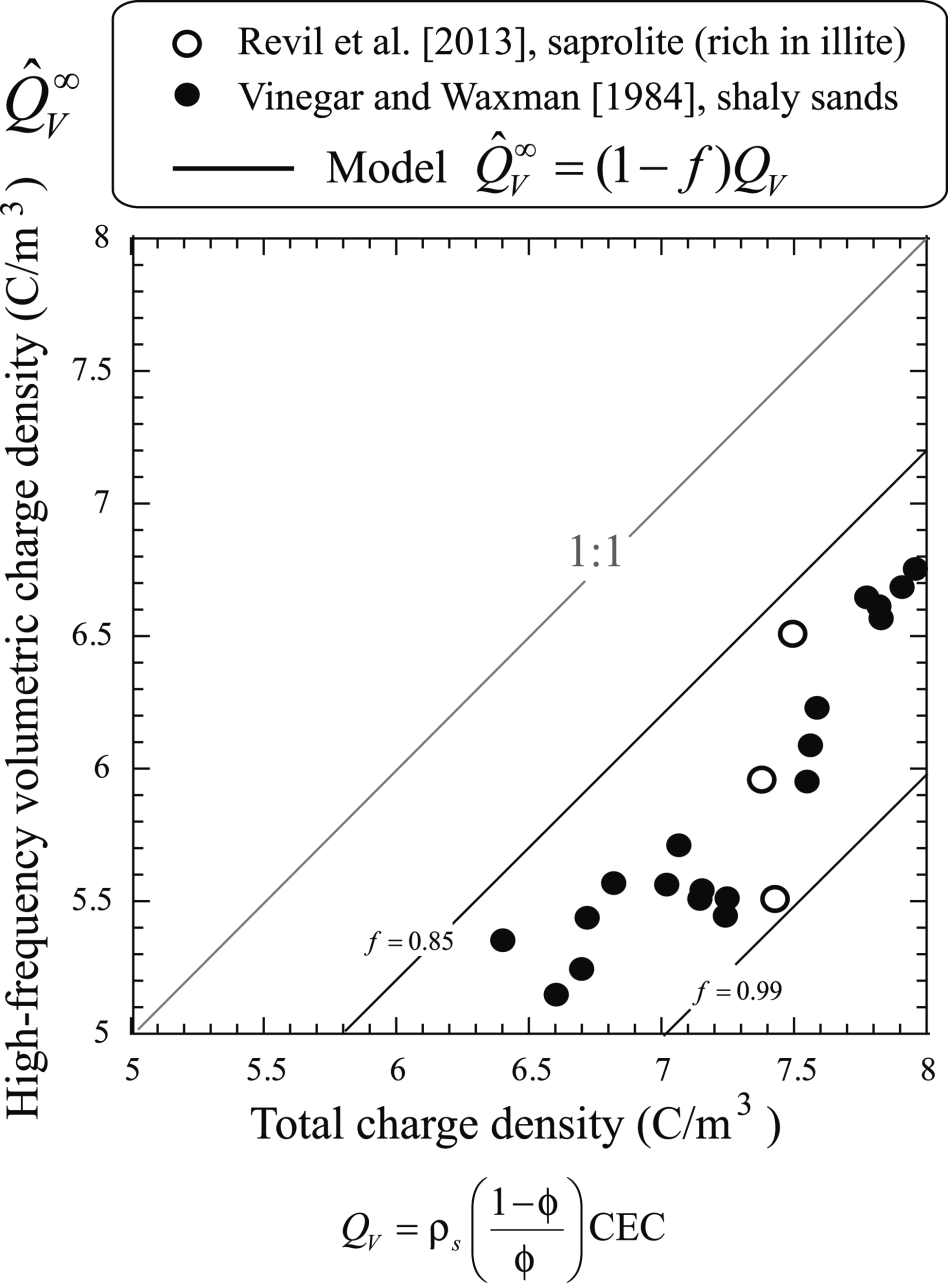
High-frequency volumetric charge density versus the total charge density. The high-frequency volumetric charge density is determined from electrical conductivity measurements at various salinities (from the surface conductivity and the formation factor) while the total charge density is determined from the porosity and CEC.

The data set of *Vinegar and Waxman* [1984] database was analyzed using the linear conductivity model described above. The results (not shown here) are actually pretty close to the results of the differential effective medium model used by *Revil* [2012, Table 1]. The factor 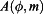 is typically comprised between 4 and 14. In Figure 5, the high-frequency charge density determined from surface conductivity is plotted as a function of the total charge density estimated from the measured CEC using a titration method. From this graph, the fraction of counterions in the Stern layer is comprised between 0.85 (85%) and 0.99 (99%). According to *Revil* [2012], the maximum partition coefficients are reached at high salinities with *f*(kaolinite) = 0.98, *f*(illite) = 0.90, and *f*(smectite) = 0.85. *Revil* [2012] provides a way to compute the salinity dependence of *f* using a simplified complexation model at the surface of the minerals.

According to Figure 5, 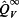 is in the range 10^5^ to 10^7^ C m^−3^ for permeability in the range 10^−16^ to 10^−12^ m^2^ [see *Vinegar and Waxman*, 1984; *Revil*, 2012]. For this permeability interval and according to Figure 3, the volumetric charge density 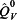 would be in the range 1–1000 C m^−3^. Therefore, for most porous media the assumption 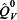 ≫ 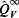 (with the exception of shales or formations/soils extremely rich in clays) is justified.

## 6. Comparison With Experimental Data

There is lack of experimental data to evaluate the accuracy and predictive power of our model with respect to the effect of both the water saturation and for the full range of frequencies covering the viscous and inertial dominated regimes. In the following two sections, we compare our model separately for (1) the frequency dependence of the streaming potential coupling coefficient showing how salinity and frequency affects this fundamental coupling parameter and (2) the effect of saturation on the low-frequency coupling coefficient.

### 6.1. Saturated Case: Effect of the Inertial Term

We first investigate the effect of salinity upon the streaming potential coupling coefficient. From equations (27) and (38) at saturation (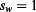) and at low frequencies (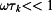), we obtain,


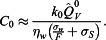
(104)

**Figure 6:**
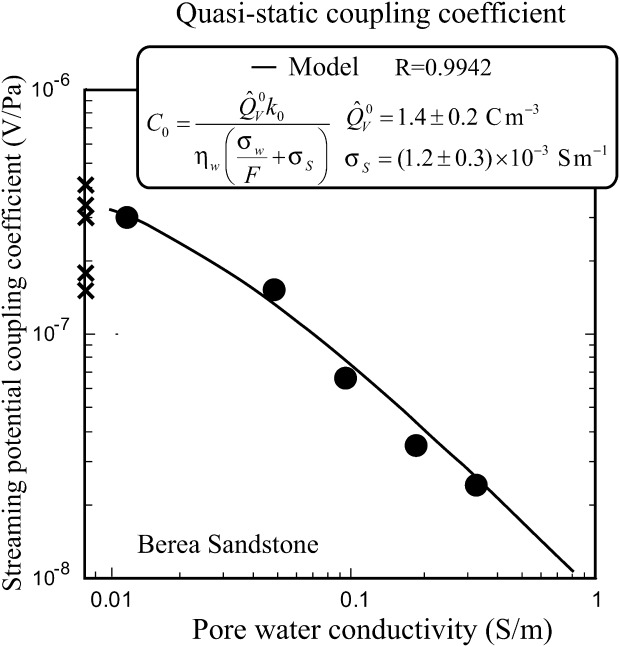
Static streaming potential coupling coefficient. The black circles correspond to measurements by *Zhu and Toksöz* [2012] (Berea sandstone, porosity 0.23, permeability 450 mD, NaCl). The crosses come from laboratory measurements by *Moore et al*. [2004] (Berea sandstone, porosity 0.19, water). The plain line corresponds to the fit the proposed model to the data of *Zhu and Toksöz* [2012] only.

We first fit the values of the static coupling coefficient displayed in Figure 6 for the Berea sandstone using equation (104)–(4). We obtain 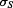 = (1.2±0.3)×10^−3^ S m^−1^ and 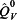 = 1.4±0.2 C m^−3^. These two values can be independently confirmed using our model: (1) The value of 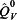 can be independently obtained by equation (98), which yields 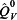 = 2.0 C m^−3^. (2) The surface conductivity can be compared to the estimate made by *Moore et al*. [2004] using electrical conductivity measurements. They found 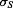 = 2.7 × 10^−3^ S m^−1^. Therefore, there is a fair agreement between the present theory and the published experimental data. From the surface conductivity (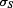 = 1.2×10^−3^ S m^−1^), the formation factor (*F* = 18), and the value of the mobility of the counterions in the diffuse layer (*β*(Na^+^, 25°C) = 5.2×10^−8^ m^2^ s^−1^ V^−1^), we can estimate the value of the high-frequency charge density 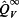 using equation (100). We obtain 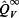 = 4×10^5^ C m^−3^. We check therefore that 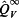 ≫ 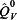 in the case of the Berea sandstone (porosity 0.23, permeability 450 mD, NaCl). This is expected because the Berea sandstone is a sandstone with pretty large pores (6–9 µm) and therefore the double layer is very thin with respect to the size of the pores.

**Figure 7:**
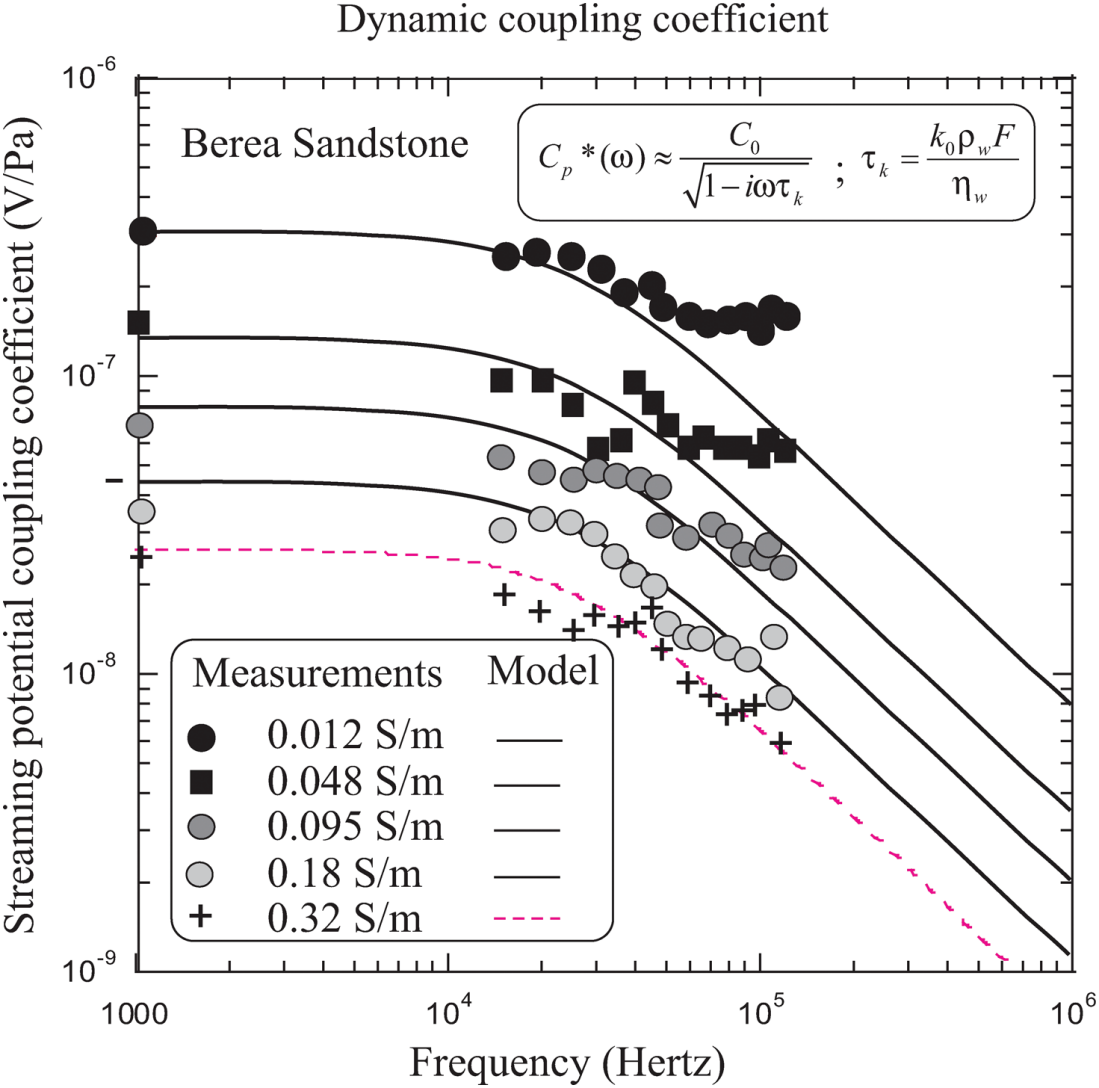
Dynamic streaming potential coupling coefficient (the value reported at 1 kHz are the static values). The data are from *Zhu and Toksöz* [2012] for the same Berea sandstone (porosity 0.23, permeability 450 mD, NaCl). The relaxation is due to the transition between the viscous-laminar flow regime at low frequency and the inertial laminar flow regime at high frequencies. Below 10 kHz, the streaming potential coupling coefficient can be considered independent on the frequency.

We used now the previous results to predict the frequency dependence of the streaming potential coupling coefficient of the Berea sandstone. In Figure 7, we compare the prediction of our model with the recent measurements of the dynamic streaming potential coupling coefficient from *Zhu and Toksöz* [2012] (the value reported at 1 kHz are actually the static values). The present model is able to reproduce these data very well up to 100 kHz for five different salinities.

### 6.2. Unsaturated and Quasi-Static Case

We can now evaluate the effect of water saturation upon the streaming potential coupling coefficient by substituting inside equations (27) and (38) the volumetric charge density, the permeability, and the electrical conductivity by their expressions as a function of saturation. At high salinity, 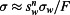, the low-frequency charge density scales as 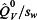, and the relative permeability scaling with the saturation is given by equation (68). We therefore obtain the following expression for the quasi-static steaming potential coupling coefficient: The quasi-static streaming potential coupling coefficient is therefore given by 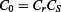, where the quasi-static streaming potential coupling coefficient at saturation is given by equation (15) and the relative coupling coefficient is given by,


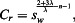
(105)

**Figure 8:**
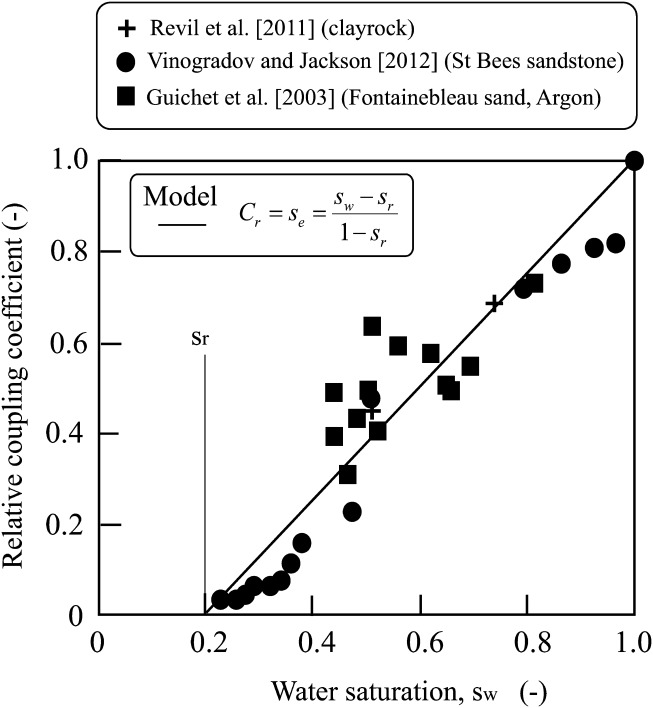
Quasi-static relative streaming potential coupling coefficient as a function of the water saturation. The plain line corresponds to the equation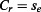 with an irreducible water saturation *s_r_* = 0.2. Data from *Revil et al*. [2012], *Vinogradov and Jackson* [2011], and *Guichet et al*. [2003].

Because the streaming potential coupling coefficient needs to be equal to zero at the irreducible water saturation, we can replace the water saturation by the effective water saturation *s_e_*.


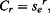
(106)

where 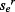 denotes the reduced water saturation and 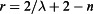. The same approach at low salinity (surface conductivity dominates) yields 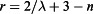. In *Revil* [2013, Appendix B], it is shown that 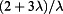 scales as (*n*+2). So at high salinity, this yields 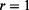. In Figure 8, we plot a number of recently reported experimental data, and we confirm that indeed *r* = 1 can be used as a good first-order approximation. At low salinity, a similar analysis yields *r* = 2.

## 7. Application to a Remediation Problem

We show an application of the presented model to the detection of the NAPL(oil) water encroachment front during the remediation of an NAPL(oil) contaminated aquifer by flooding the aquifer with water (such remediation can be enhanced also with the use of surfactants, e.g., *Mercier and Cohen* [1990], *Pope and Wade* [1995], *Londergan et al*. [2001]). We will use subscript “o” to characterize the properties of oil.

### 7.1. Simulation of Water Flooding of an NAPL-Contaminated Aquifer

We follow the following two steps to simulate the remediation of a NAPL(oil)-contaminated aquifer.

**Figure 9:**
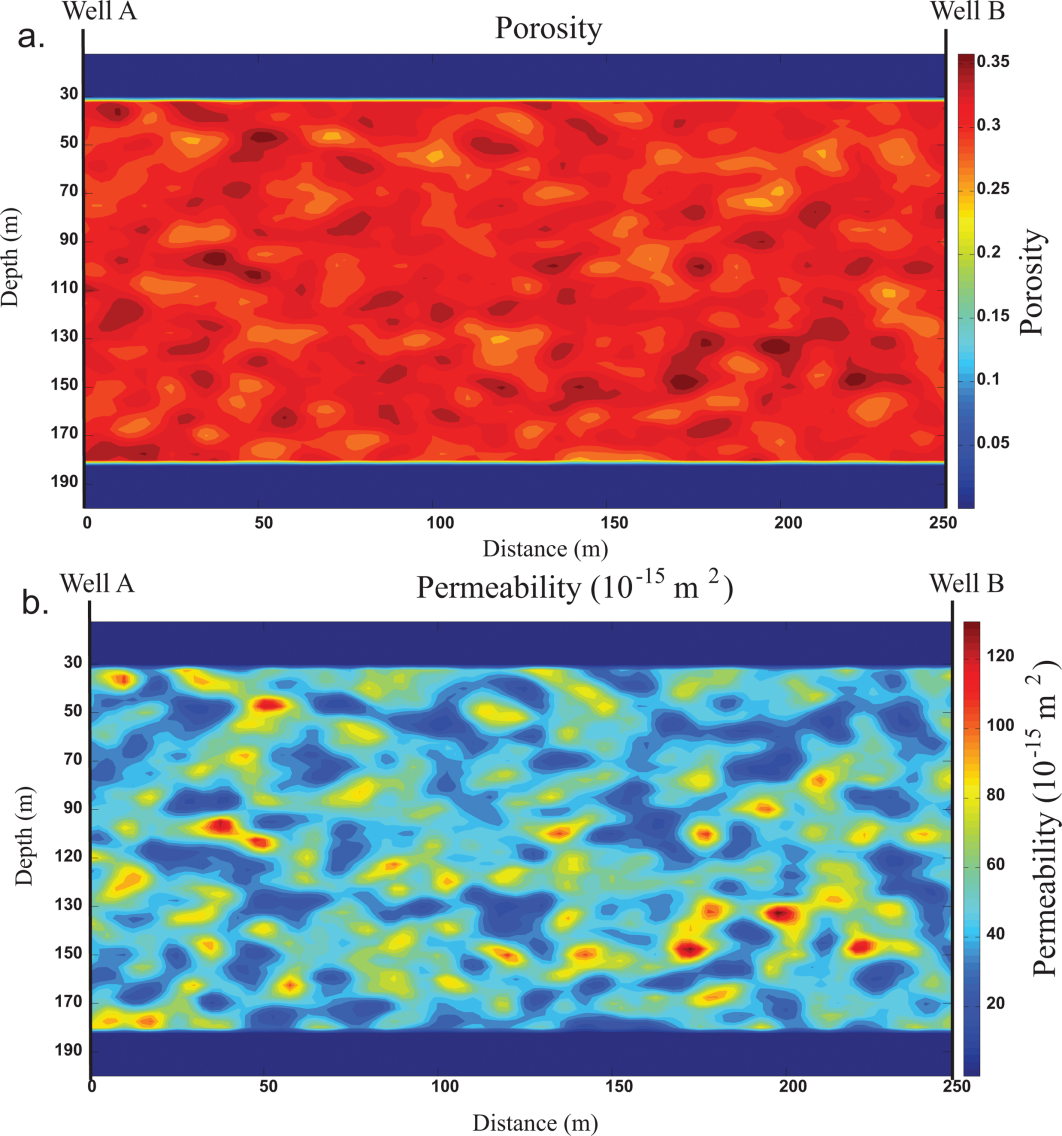
Porosity and permeability map of an NAPL (oil) contaminated aquifer between two wells for a water-flood simulation.

Step 1. We used the approach developed in *Karaoulis et al*. [2012] to generate a 2-D heterogeneous aquifer in terms of porosity and permeability (Figure 9). A random field for the clay content was generated with the SGeMS library [*Stanford University*]. We used an isotropic semivariogram to compute the clay content distribution (see *Karaoulis et al*. [2012, Appendix A]). The porosity and permeability were then computed according to the petrophysical model defined by *Revil and Cathles* [1999]. This heterogeneous aquifer is assumed to be initially saturated with 75% of light motor oil, resulting from an oil spill. The initial water saturation of the aquifer is therefore 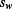 = 0.25, which corresponds to the irreducible water saturation 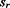. This aquifer is located between two wells: Wells A and B. Well B is located 250 m away from Well A. The reference position, O(−80,30) for the coordinate system is located at the upper left corner of this domain.

**Figure 10:**
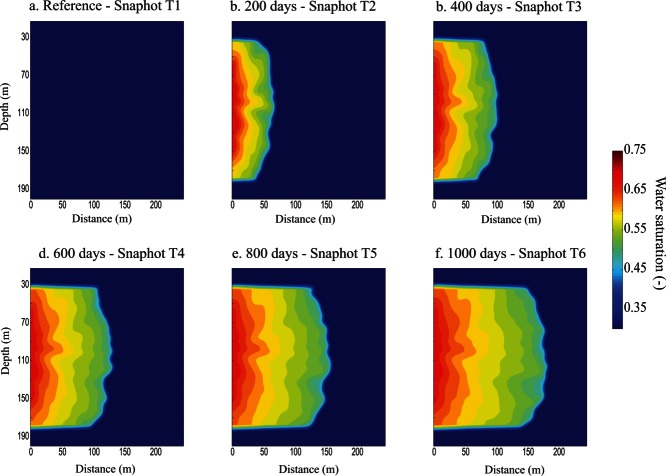
Six snapshots showing the evolution of the water saturation *s_w_* over time in a 150-m-thick NAPL(oil) contaminated aquifer. The initial water saturation in the aquifer is equal to the irreducible water saturation *s_r_* = 0.25 (which correspond to a NAPL saturation of 0.75). In this study, the NAPL (oil) is considered to be the nonwetting phase.

Step 2. Water flooding of this aquifer is simulated in 2.5D by injecting water in Well A (constant injection rate) and removing the NAPL(oil) in Well B (constant pressure condition). The computations are done in two-phase flow conditions following the same equations as in *Karaoulis et al*. [2012, Appendix A]. The properties of the NAPL(oil) and water are reported in Table 3. We use a relatively low viscosity for the NAPl(oil) as usually the injected water is heated to decrease the NAPL(oil) viscosity. Also the NAPL is assumed to be the nonwetting phase in this numerical experiment. This is realistic only just after an oil spill for instance. Indeed, after a period of few years, the wettability (surface tension) of the oil can change because bacteria produced, for instance, biopolymers bridging the oil molecules to the surface of the grains. This effect is not accounted for here. After the simulations, we display six snapshots (T1–T6) of the oil and water saturations (Figure 10).

**Table 3:** Material Properties Used in the SE Forward Model

Parameter	Value	Units	Reference
*ρ_s_*	2650	kg m^−3^	*Mavko et al*. [1999]
*ρ_w_*	1000	kg m^−3^	*Mavko et al*. [1999]
*ρ_o_*	900	kg m^−3^	*Karaoulis et al*. [2012]
*K_s_*	36.5	GPa	*Mavko et al*. [1999]
*K_fr_*	18.2	GPa	*Mavko et al*. [1999]
*G*	13.8	GPa	*Mavko et al*. [1999]
*K_w_*	2.25	GPa	*Jardani et al*. [2010]
*K_o_*	1.50	GPa	*Charoenwongsa et al*. [2010]
*η_w_*	1×10^−3^	Pa s	*Jardani et al*. [2010]
*η_o_*	50×10^−3^	Pa.s	Light motor oil

### 7.2. Simulation of the SE Problem

For each of the snapshots shown in Figure 10, we simulated a SE acquisition between the two wells. The simulation of the SE problem is also done in two phases as described below. Because this problem is formally different from the unsaturated case discussed above (two-phase flow problem versus unsaturated problem), we need to accommodate somehow this issue as discussed below.

Step 1. This step concerns the modeling of the propagation of the seismic waves between the two wells. We use material properties values given in Table 3 for the computation of the seismic properties. The bulk modulus of the fluid is related to the NAPL(oil) saturation by using Wood formula as discussed in section 3.1. above [e.g., *Rubino and Holliger*, [2012]:


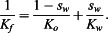
(107)

where *K_o_* and *K_w_* denote the bulk moduli of NAPL(oil) and water, respectively. The shear modulus is independent on the saturation because none of the two fluids sustain shear stresses. The bulk modulus of the fluid is given by equation (107), and the density and viscosity of the fluid is given by,


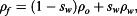
(108)


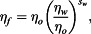
(109)

where 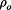 and 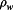 denote the density of the NAPL(oil) and water, respectively, and 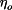 and 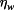 denote the dynamic viscosity of oil and water, respectively. The difference of fluid pressure between the two phases is controlled by the capillary pressure curve, which is given by the same capillary pressure curve used to simulate the two-phase flow problem (see section 7.1 and *Karaoulis et al*. [2012, Appendix A]).

**Figure 11:**
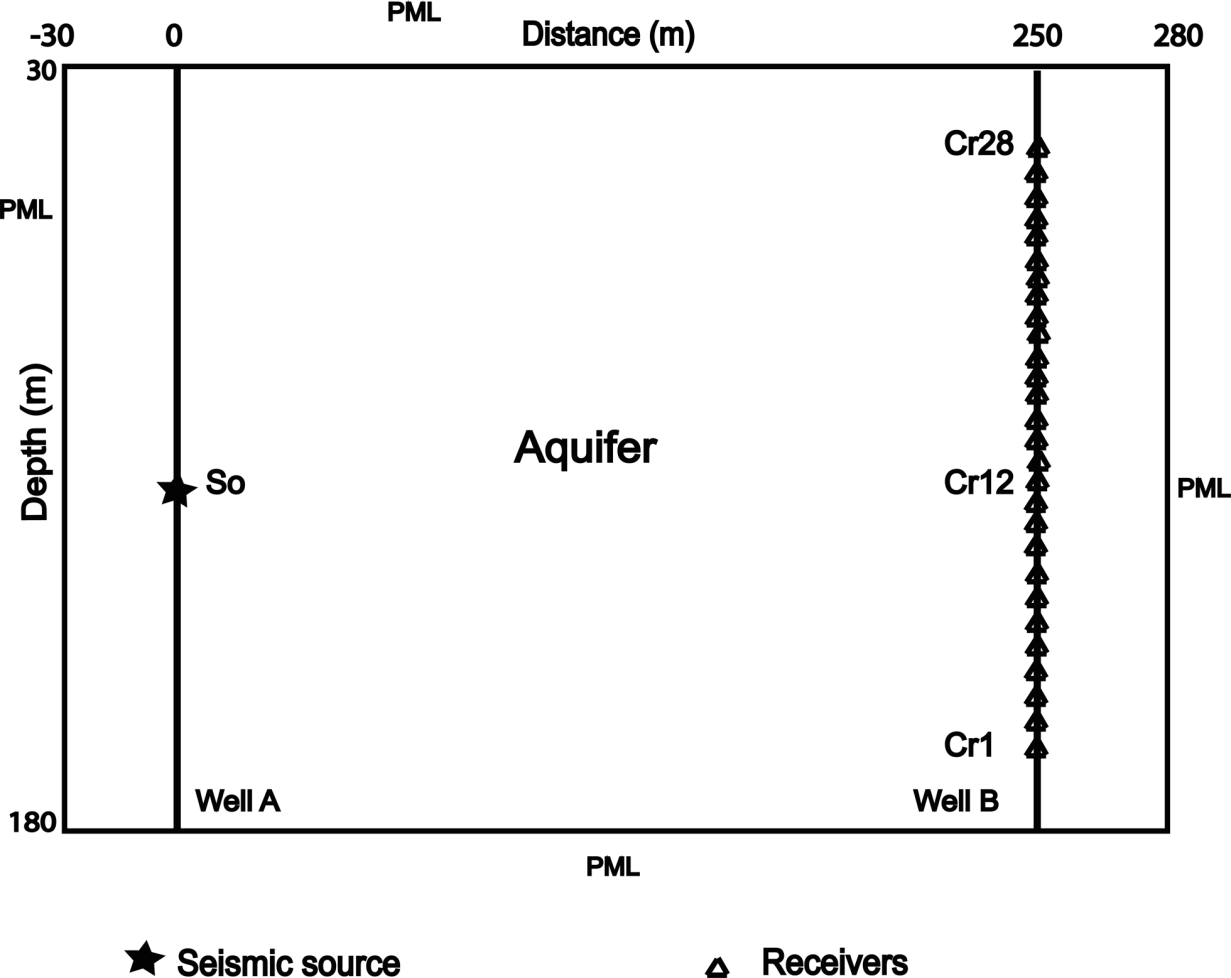
Sketch of the domain used for the modeling. The domain is a 350 m × 350 m square. The injection well (Well A) is located at position *x* = 0 m. The seismic source is located in this well at *S*_0_ (0 m, 116 m). The 71 receivers (Cr1–Cr71) are located in the production Well B located at *x* = 250 m. The discretization of the domain comprises a finite element mesh of 125 × 125 rectangular cells. Perfectly matched layer (PML) adsorbing boundary conditions are used at the borders of the domain to simulate the wave propagation problem with open boundaries.

The geometry of the model used for the computation of the seismic waves is shown in Figure 11. The seismic sources is an explosive-like source located at position So in Well A (Figure 11). The receivers comprise 28 pairs of seismic stations and electrodes (noted as Cr1–Cr28), which are located in Well B. The separation between these receivers is equal to 4 m.

We first solve the poroelastodynamic wave equations in the frequency domain, taking into account the variable saturation of the water phase. We use the (**u**, *p*) formulation of section 3.2 above (see *Jardani et al*. [2010] for going to the exact form of the partial differential equations). We use the same approach as in *Rubino and Holliger* [2012]. We use the multiphysics modeling package Comsol Multiphysics 4.2a and the stationary parametric solver PARDISO to solve the resulting partial differential equations [*Schenk and Gärtner*, 2004, 2006; *Schenk et al*., 2007, 2008]. The problem is solved as follow: (i) we first compute for the poroelastic and electric properties distribution for the given porosity, fluid permeability, and saturation distribution of the NAPl(oil) and water phases, then (ii) we solve for the displacement of the solid phase **u** and the pore fluid pressure *p* in the frequency domain. The solution in the time domain is computed by using an inverse-Fourier transform of the solution in the frequency wave number domain (see *Jardani et al*. [2010] and Araji et al. [2012] for details regarding the numerical procedure).

We first solve the poroelastodynamic wave equations in the frequency domain, taking into account the variable saturation of the water phase. We use the (**u**, *p*) formulation of section with *m* = *n* = 2 and a surface conductivity 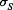 = 0.01 S m^−1^ while the conductivity of the pore water is setup at 0.1 S m^−1^. The charge density 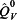 is determined from equation (98) and the distribution of the permeability (see Figure 10). The source current density is determined with equations (106) and (107) and is therefore consistent with the data shown in Figure 8 and the solution for the displacement of the solid phase and the fluid pressure. Finally, the electrical potential distribution is obtained by solving a Poisson equation for the electrical potential.

### 7.3. Results

**Figure 12:**
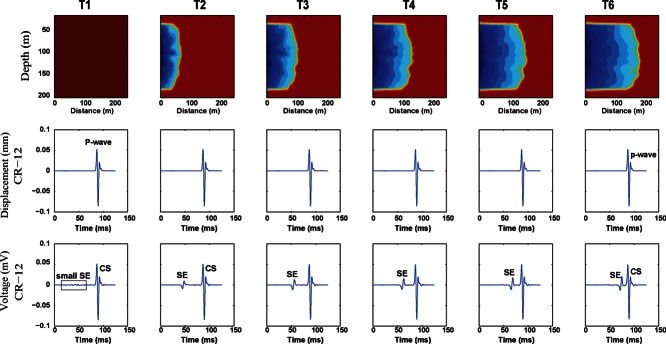
Evolution of the seismic displacement and the associated electric potential time series from receiver point Cr12 due to changes in the position of the oil-water encroachment front during snapshots T1–T6. The arrival of the P wave is well identified in the seismic time series. SE denotes the SE conversions occurring at any electrical and mechanical heterogeneity in the aquifer (especially at the oil-water encroachment front) while CS denotes the coseismic electrical field associated with the P wave.

The evolution of the seismic displacement and the electrical potential time series recorded at station Cr12 for each saturation profile (T1–T6) are shown in Figure 12. The seismic displacements are generated from the seismic source, which allows only for generation of P waves. In this case, with the porosity distribution displayed in Figure 9 and with the water saturation variations shown in Figure 10, the average P-wave velocity of profile T1–T6 is about 4800 m s^−1^. Therefore, the P-wave arrivals in Well B is roughly the same at the snapshots T1–T6 (Figure 12). Figure 12 shows that SE conversions occur for each snapshot. These conversions always arrive earlier than the coseismic electrical field associated with the arrival of the P wave. They also arrive later and later as the water front progresses toward Well B. There is therefore a clear conversion mechanism at the NAPL(oil)/water encroachment front for each of the five snapshots T2–T6. For snapshot T1, since there is no saturation contrast, we do not see any strong SE conversions. That said, there are still some small SE conversions taking place at the heterogeneities in the aquifer.

**Figure 13:**
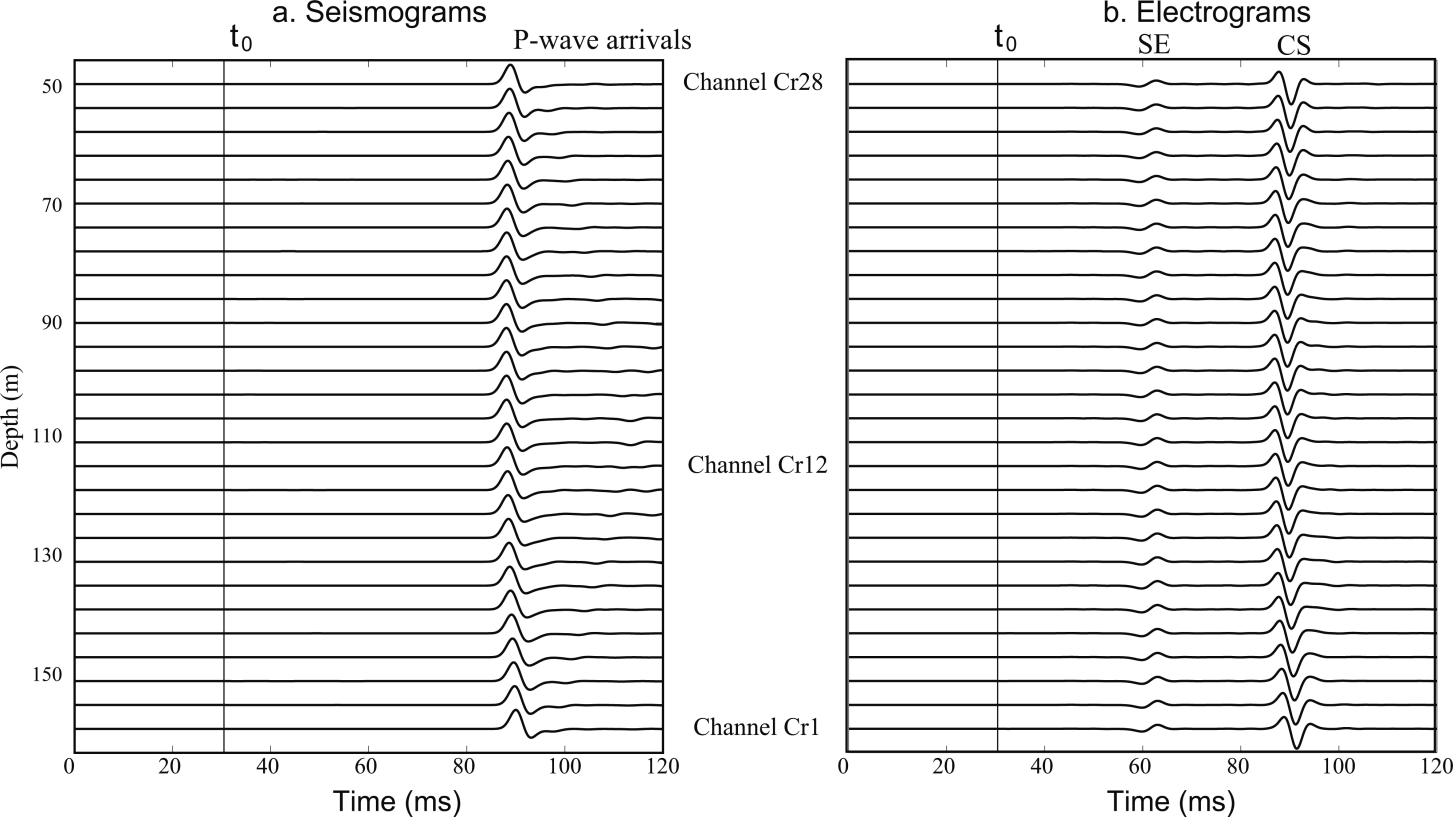
Seismogram and electrograms from implementing saturation profile T4. (a) The seismograms reconstructed by the geophones show the *P*-wave propagation from the seismic source in Well A to recording Well B. (b) The electrograms show the coseismic electrical potential field associated with the P-wave (CS) and the SE conversions with a smaller amplitude and the same time arrival.

**Figure 14:**
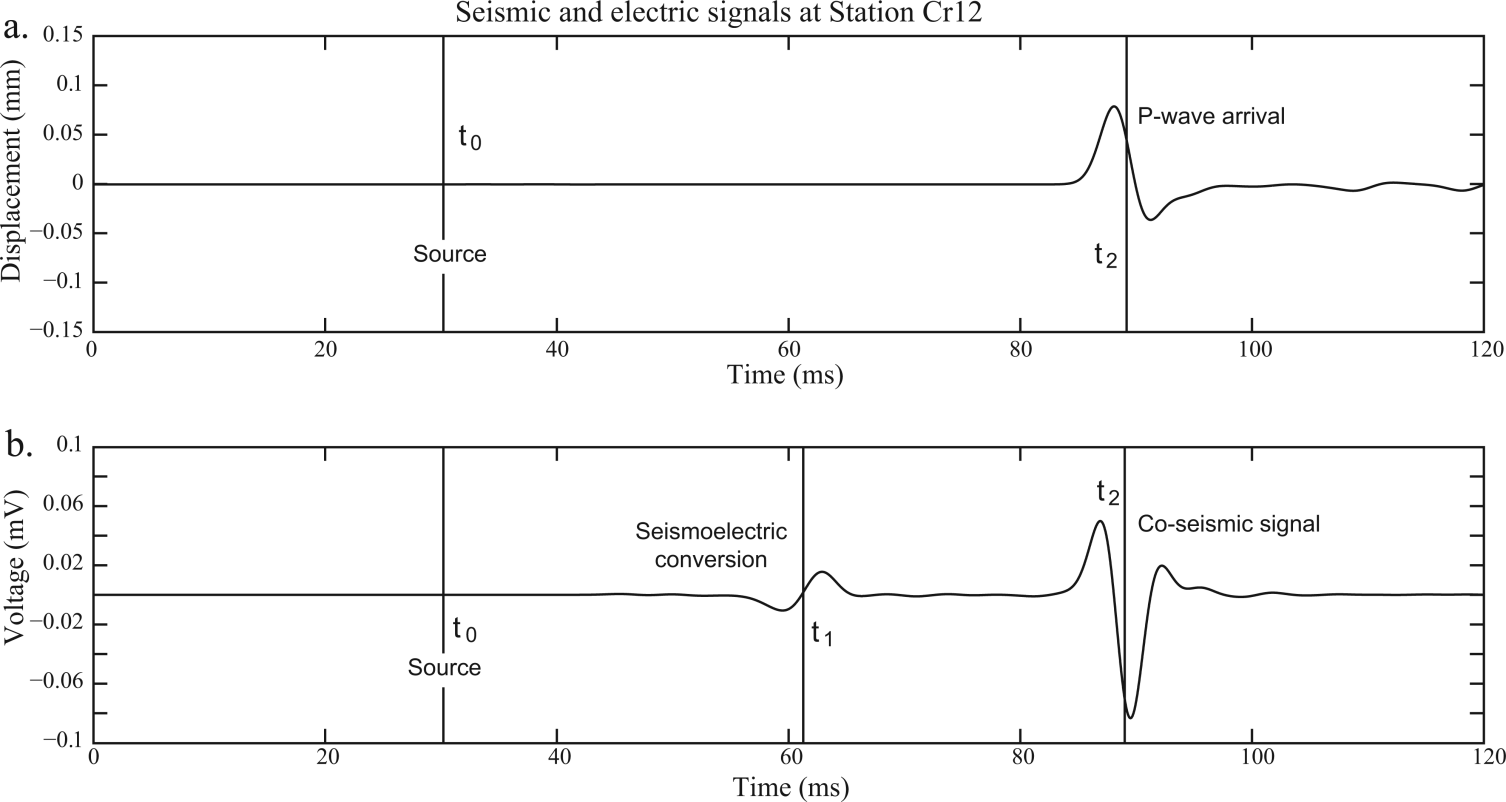
Seismogram and electrogram at receiver Cr12 (see Figure 12). Here *t*_0_ denoted the time of source ignition or the delay time (30 ms). The arrival times of the SE signals and the coseismic disturbance associated with the P wave are denoted as *t*_1_ and *t*_2_, respectively. The strongest signal on the electrogram corresponds to the coseismic disturbance associated with the P-wave propagation (see Figure 13).

Taking the saturation profile T4 into the model, we show that both SE conversion generated at the NAPL(oil) water encroachment front and coseismic (CS) electrical signals are shown in all receiving stations Cr1–Cr28 (Figure 13). Figure 14 shows the seismic displacement and the electrical time series for station Cr12 with information on the delay time *t*_0_, SE conversion at time *t*_1_, and the similarities of coseismic and P-wave arrival time *t*_2_.

In terms of amplitudes, the type of signal measured here is easily recordable in the laboratory and in the field through stacking. Dupuis, Butler, and colleagues have developed methods to improve the signal-to-noise ratio in SE investigations, and they demonstrated that there are no serious issues in measuring SE conversions in field conditions [*Dupuis and Butler*, 2006; *Dupuis et al*., 2007, 2009]. In conclusion, we see that our numerical model implies that the NAPL(oil)/water encroachment front can be detected through SE measurements.

## 7. Concluding Statements

The following conclusions have been reached:

(1) We have developed the first SE/electroseismic model in unsaturated conditions, which is valid whatever the thickness of the double layer with respect to the size of the pores. This model is based on the recently introduced concept of effective charge density that can be dragged by the flow of the pore water. This effective charge density can be related directly to the permeability, thereby avoiding the introduction of parameters that can be difficult to assess independently for field applications.

(2) The model has been tested with respect to experimental data as a function of the frequency at full saturation and as a function of the saturation at low frequencies. There is a need to test the model at partial saturation in the full range of frequencies covering the viscous-laminar and inertial laminar flow regimes.

(3) The model can be easily modified to account for two-phase flow conditions for applications to remediation problems of NAPL/DNAPL plumes. We described a numerical model related to an application that is relevant of the early NAPL(oil) contamination of a clayey sand aquifer. We have shown that the oil water encroachment front can be remotely detected using cross-well SE conversions. Therefore, this opens the door to a broad range of applications to monitor and image remotely changes in the water saturation in the vadose zone and the monitoring of NAPLs and DNAPLs plumes.

## Appendix A: Frequency Dependence of the Volumetric Charge Density

We first define the effective (moveable) charge density in quasi-static flow conditions. We write the macroscopic charge density (charge per unit pore volume, in C m^−3^) that is dragged by the flow of the pore water as . This charge density is given by,

(A1)

(A2)

where the brackets denote a pore volume averaging,  denotes the local charge density in the pore space (in C m^−3^),  denotes the local instantaneous velocity of the pore water with respect to the solid phase (in m s^−1^), **x** is a local position in the pore space of the material, and  is an elementary volume around point . Equation (A1) is valid whatever the size of the diffuse layer with respect to the size of the pores. The macroscopic source current density  (called the streaming current density) is related to the local current density  defined with respect to the deformation of the solid phase by,

(A3)

(A4)

(A5)

(A6)

This is showing how the charge density  is not a static charge density but a dynamic one associated with the average of the pore water velocity. This is why this effective charge density is very sensitive to the permeability of the porous material as shown in Figure 3.

We continue now with the thin-double layer theory of Pride. In this limit, our model has to be compatible with his theory based on the zeta potential. Taking the low-frequency limit of *Pride* [1994, equation (237)] and dropping the second-order terms in the thin double layer limit, the cross-coupling term  is given by,

(A7)

and in the present context, the low-frequency coupling term is given in saturated conditions by,

(A8)

In the thin double-layer theory based on the excess charge density, the frequency-dependent cross-coupling term  is related to the frequency-dependent permeability and charge density by (in saturated conditions),

(A9)

In addition, the frequency dependence of the permeability has already been established and is given by,

(A10)

From equations (A7)–(A10), saturated conditions, in the thin double-layer limit and in the low-frequency limit, the frequency dependent volumetric charge density is given by

(A11)

Now in the high-frequency limit, the limit of the volumetric charge density  is given by,

(A12)

A simple way to connect simply and smoothly equations (A11) and (A12) is to use the following function,

(A13)

Equation (A13) has been established in the thin double-layer limit. For the thick double-layer case,  (the same density of charges is dragged along with the pore water flow independently on the frequency) and . In the thick double-layer case, there is no frequency dependence of the volumetric charge density as discussed in detail in *Revil and Linde* [2006]. Equation (A13) provides therefore the correct solution in this case too. In the thin double-layer approximation (see Figure 2b),  (see examples in *Jougnot et al*. [2012] and section 5 of the main text). In the thick double-layer approximation (see Figure 2a),  (all the counterions of the diffuse layer are dragged by the flow whatever the frequency) and therefore the frequency-dependent excess charge density  is given by,

(A14)

The charge density  can be determined from the clay composition, the CEC of the different clay minerals, and their mass fraction (see section 5.2 of the main text).
